# Development of an *o*-COSAN Ion-Pair Complex-Modified PVC Membrane for Microconductometric Glyphosate Detection

**DOI:** 10.3390/mi17080901

**Published:** 2026-07-27

**Authors:** Youssef O. Al-Ghamdi, Amani Chrouda, Nicole Jaffrezic-Renault, Hamdi Ben Halima

**Affiliations:** 1 Department of Chemistry, College of Science Al-Zulfi, Majmaah University, Al-Majmaah 11952, Saudi Arabia; yo.alghamdi@mu.edu.sa (Y.O.A.-G.); amain.c@mu.edu.sa (A.C.); 2 Centre National de la Recherche Scientifique (CNRS), Institut UTINAM (UMR 6213), Université Marie et Louis Pasteur, F-25000 Besançon, France

**Keywords:** glyphosate, conductimetric sensor, ion-pair complex, environmental monitoring

## Abstract

Glyphosate is among the most widely used herbicides worldwide, and its extensive application has led to increasing concerns regarding environmental contamination and potential risks to human health. The persistence of this compound in soil and aquatic environments has created an urgent demand for analytical methods that are rapid, reliable, and economically feasible. In the present study, a microconductometric sensing platform was developed for glyphosate determination using a PVC liquid membrane incorporating an [*o*-COSAN]⁻/glyphosate ion-pair complex deposited onto interdigitated electrodes. The proposed sensor provided a linear analytical response over the concentration range of 1.0 × 10⁻^5^ to 2.5 × 10⁻^3^ M, with a detection limit of 4 μM. The device also exhibited excellent analytical performance, with reproducibility and repeatability values of 3% and 8% (RSD), respectively. Furthermore, the sensor maintained stable performance for more than three months and showed a high degree of selectivity toward glyphosate when evaluated against potential interfering compounds, including AMPA and carbofuran. These results demonstrate the potential of the proposed sensing platform as a simple, sensitive, and cost-effective tool for glyphosate monitoring in environmental samples.

## 1. Introduction

Glyphosate (N-(phosphonomethyl)glycine) is the most widely used non-selective herbicide worldwide owing to its broad-spectrum weed control efficiency and relatively low production cost. It is extensively applied in agriculture, forestry, and urban vegetation management [1,2]. Its herbicidal activity results from the inhibition of 5-enolpyruvylshikimate-3-phosphate synthase (EPSPS), an essential enzyme of the shikimate metabolic pathway responsible for the biosynthesis of aromatic amino acids in plants and many microorganisms [3]. Since this metabolic pathway is absent in mammals, glyphosate was initially regarded as having relatively low toxicity. However, its intensive and widespread use has raised increasing concerns regarding its environmental persistence and potential impacts on human health [4]. However, in 2005, the Food and Agriculture Organisation (FAO) reported that glyphosate and its major metabolite, aminomethylphosphonic acid (AMPA), are of potential toxicological concern, mainly as a result of accumulation of residues in the food chain. The FAO further states that the dietary risk of glyphosate and AMPA is unlikely if the maximum daily intake of 1 mg kg^−1^ body weight (bw) is not exceeded [5].

The extensive and repeated application of glyphosate has resulted in its widespread occurrence in various environmental compartments, including surface water, groundwater, soils, food products, and biological samples [5]. Consequently, the development of reliable analytical methods for its determination has become increasingly important. Nevertheless, the analysis of glyphosate remains challenging because of its physicochemical properties, particularly its high polarity, strong affinity for aqueous media, and the absence of chromophoric groups. These characteristics limit direct detection by many conventional analytical techniques and often require derivatization or specialized analytical procedures to achieve accurate quantification [6]. Accurate, sensitive, and rapid detection methods are therefore essential for monitoring environmental contamination, assessing human exposure, and enforcing regulatory limits.

Conventional analytical methods, including high-performance liquid chromatography (HPLC) and gas chromatography coupled with mass spectrometry (GC-MS), provide excellent sensitivity and selectivity for glyphosate determination. However, their routine application is often limited by labor-intensive sample preparation, derivatization procedures, the need for expensive instrumentation, and the requirement for highly trained operators [7,8,9,10,11,12,13,14]. To overcome these limitations, alternative sensing platforms have attracted increasing interest, particularly electrochemical sensors, which offer advantages such as portability, low cost, and potential for real-time field analysis.

Recent advances in nanotechnology and the development of functional materials have greatly expanded the possibilities for glyphosate sensing. Among the various recognition strategies, molecularly imprinted polymers (MIPs) have emerged as attractive materials because they contain artificial binding sites specifically tailored for the target molecule. Their high recognition capability, combined with electrochemical transduction, enables sensitive and selective detection even in complex environmental matrices. For instance, an electrochemical sensor incorporating a molecularly imprinted polymer has been successfully employed for glyphosate determination using electrochemical impedance spectroscopy (EIS), demonstrating the effectiveness of this approach for environmental monitoring applications [15].

To further improve sensor performance, conductive nanomaterials have been integrated into MIP-based systems. For instance, an electrochemical sensor based on molecularly imprinted polypyrrole nanotubes exhibited high selectivity toward glyphosate with a detection limit of approximately 1.94 ng mL⁻^1^, demonstrating the advantages of nanostructured conductive polymers for signal amplification and improved analytical performance [16]. Similarly, the incorporation of graphene-based materials has significantly enhanced electron transfer properties and detection sensitivity. Reduced graphene oxide-modified electrodes have been successfully used for the electrochemical detection of glyphosate, enabling detection at very low concentrations in environmental samples [17]. Whereas MIP-based sensors present high sensitivity and good selectivity, the range of detection is limited by the adsorption capacity of the MIP; moreover, a desorption step is required for liberating the fingerprints in the MIP, before its reuse, which could limit the analysis throughput and the sensor shelf lifetime.

Recent studies have explored advanced nanocomposite materials and hybrid sensing platforms to further improve analytical performance. A voltammetry sensor, based on Zn porphyrin, led to a very low detection limit of 4 nM [18]. Portable voltammetric sensing platforms based on laser-induced graphene have been developed for rapid glyphosate detection in water samples, highlighting the potential of scalable and low-cost materials for field-deployable environmental monitoring systems [19].

Only one potentiometric glyphosate sensor was published. It is based on the inhibition of urease by glyphosate, which limits the production of ammonium ions when urea is injected. The obtained detection limit is 3 µM (0.5 ppm) [20].

**Figure 1:**
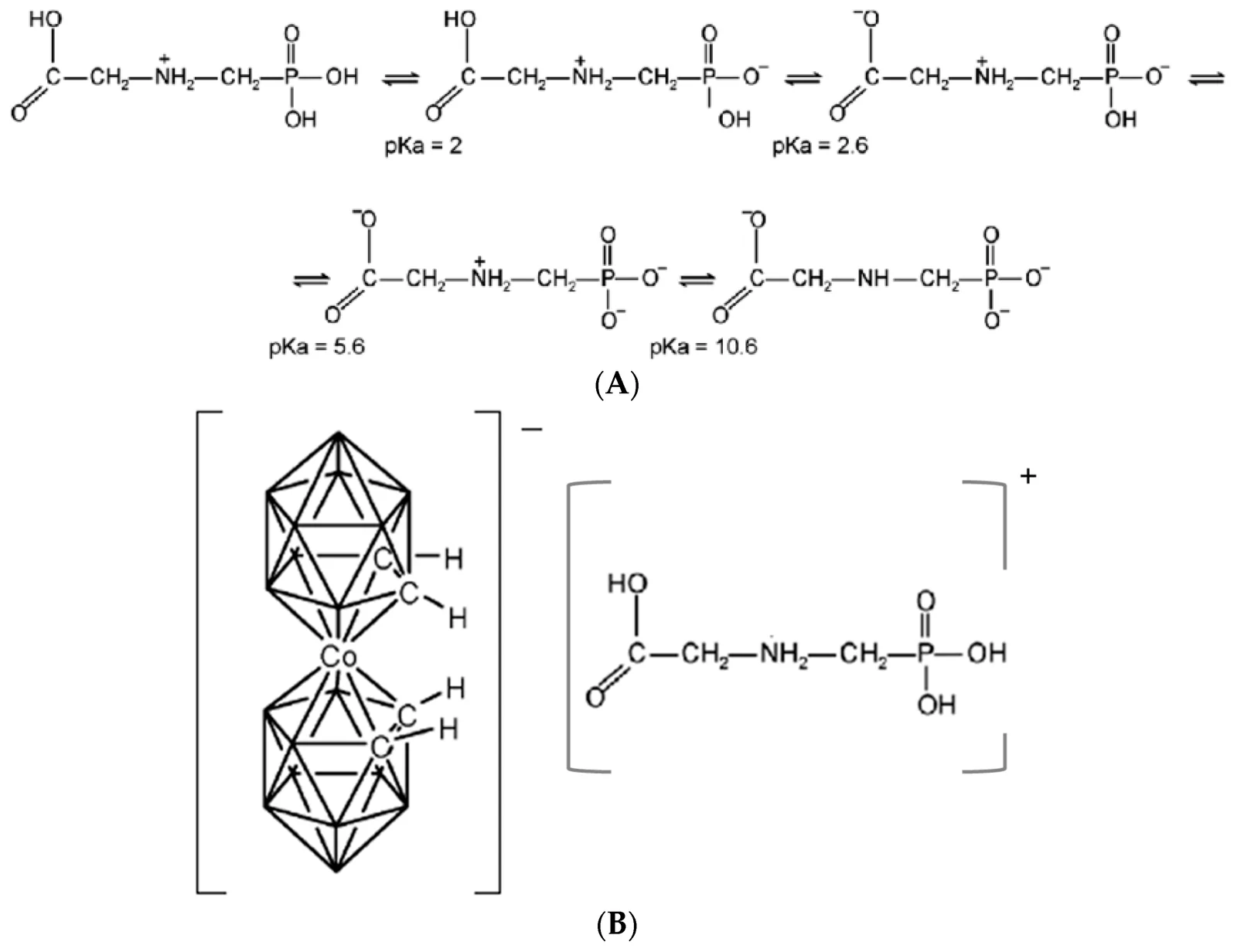
(**A**) Different forms of glyphosate molecules as a function of pH; (**B**) molecular structure of [*o*-COSAN]⁻/glyphosate ion-pair complex.

The predominant chemical form of glyphosate varies with the pH of the medium, as illustrated in Figure 1. At pH values below 2, the amino group is protonated, resulting in a positively charged glyphosate species. Under these conditions, the negatively charged [*o*-COSAN]⁻ anion readily associates with protonated glyphosate through ion-pair formation. This interaction has previously been exploited in PVC membrane-based potentiometric sensors employing *o*-COSAN ion-pair complexes for the determination of compounds such as sulfonamides, amphetamine, and enantiomers [21,22,23]. These sensors typically achieved detection limits on the order of 10⁻^6^ M.

In contrast, our multi-use conductimetric sensor platform addresses these limitations by allowing repeated measurements without compromising sensitivity or selectivity. The reusability of the system reduces the overall operational cost, minimizes environmental impact, and enables integration into automated or continuous monitoring setups. Furthermore, the robustness of the sensor materials ensures stable performance over multiple cycles, offering a practical and sustainable alternative to disposable sensing technologies. By combining high analytical performance with operational durability, our sensor system demonstrates a clear advantage over single-use platforms in terms of cost-effectiveness, environmental sustainability, and long-term usability in real-world scenarios. In this context, the present study introduces—for the first time—the use of a micro-conductometric sensor with a PVC liquid membrane for the detection of glyphosate. The sensor’s electrical response enables real-time detection of the inclusion process. Furthermore, the simplicity and scalability of conductimetric systems make them ideal candidates for integration into portable, wearable devices, enabling continuous environmental monitoring and enhancing accessibility for non-specialist users. Taken together, these technological developments underscore the growing need to address the increasing requirements for environmental monitoring, regulatory control, and food quality assessment.

## 2. Materials and Methods

### 2.1. Chemicals

Analytical-grade glyphosate, aminomethylphosphonic acid (AMPA), carbofuran, hydrochloric acid, potassium dihydrogen phosphate, tetrahydrofuran (THF), poly(vinyl chloride) (PVC), and bis(2-ethylhexyl) sebacate (DOS) were obtained from Merck (Saint-Quentin-Fallavier, France). The [*o*-COSAN]⁻ compound was supplied by Katchem spol. s r.o. (Prague, Czech Republic). Ultrapure water (resistivity > 10 MΩ·cm) was used for the preparation of all aqueous solutions.

### 2.2. Synthesis of the [o-COSAN]⁻/Glyphosate Ion-Pair Complex

The [*o*-COSAN]⁻/glyphosate ion-pair complex was synthesized by mixing equimolar amounts of glyphosate and sodium o-cobaltabisdicarbollide (Na[*o*-COSAN]). Initially, 0.2 mmol of glyphosate was dissolved in 25 mL of 3 M hydrochloric acid under continuous stirring until a clear solution was obtained. Subsequently, 0.2 mmol of Na[*o*-COSAN], previously dissolved in 10 mL of 3 M HCl, was slowly introduced into the glyphosate solution. The immediate formation of an orange precipitate indicated the formation of the ion-pair complex. The resulting precipitate was separated by vacuum filtration using a Büchner funnel, thoroughly washed with 0.1 M hydrochloric acid to remove residual impurities, and then rinsed with ultrapure water. Finally, the solid product was dried under reduced pressure (0.1–0.01 mmHg) at room temperature for 2 h, yielding the purified [*o*-COSAN]⁻/glyphosate complex for subsequent characterization and membrane preparation.

The structural features of the synthesized ion-pair complex were investigated by Fourier-transform infrared (FTIR) spectroscopy. Infrared spectra were acquired using a Bruker Vertex 70 FTIR spectrometer equipped with a deuterated triglycine sulfate (DTGS) detector and a Platinum attenuated total reflectance (ATR) accessory fitted with a diamond crystal. Each spectrum was recorded over the selected spectral range by averaging 128 scans at a spectral resolution of 4 cm⁻^1^, ensuring a satisfactory signal-to-noise ratio and reliable identification of the characteristic functional groups of the ion-pair complex.

Mechanical Profilometry: The thickness (T) of the membrane was assessed using a stylus (2.5 μm)-based mechanical probe profiler Alpha-Step IQ from KLA Tencor (Milpitas, CA, USA). Measurement of thickness was conducted over a scan length of 14,583 μm at a speed of 90 μm/s. The reported values for thickness and roughness represent the averages of at least 3 measurements taken from different locations on the sample.

The morphological characteristics of the membrane surface were studied using high-resolution scanning electron microscopy (SEM, MIRAN3 TESCAN, Brno, Czech Republic) operated at an electron beam energy of 7 keV.

The VigiZMeter conductometer developed by Covarians (Gif-sur-Yvette, France) was used for conductance measurements.

### 2.3. Microconductimetric Chip Preparation

**Figure 2:**
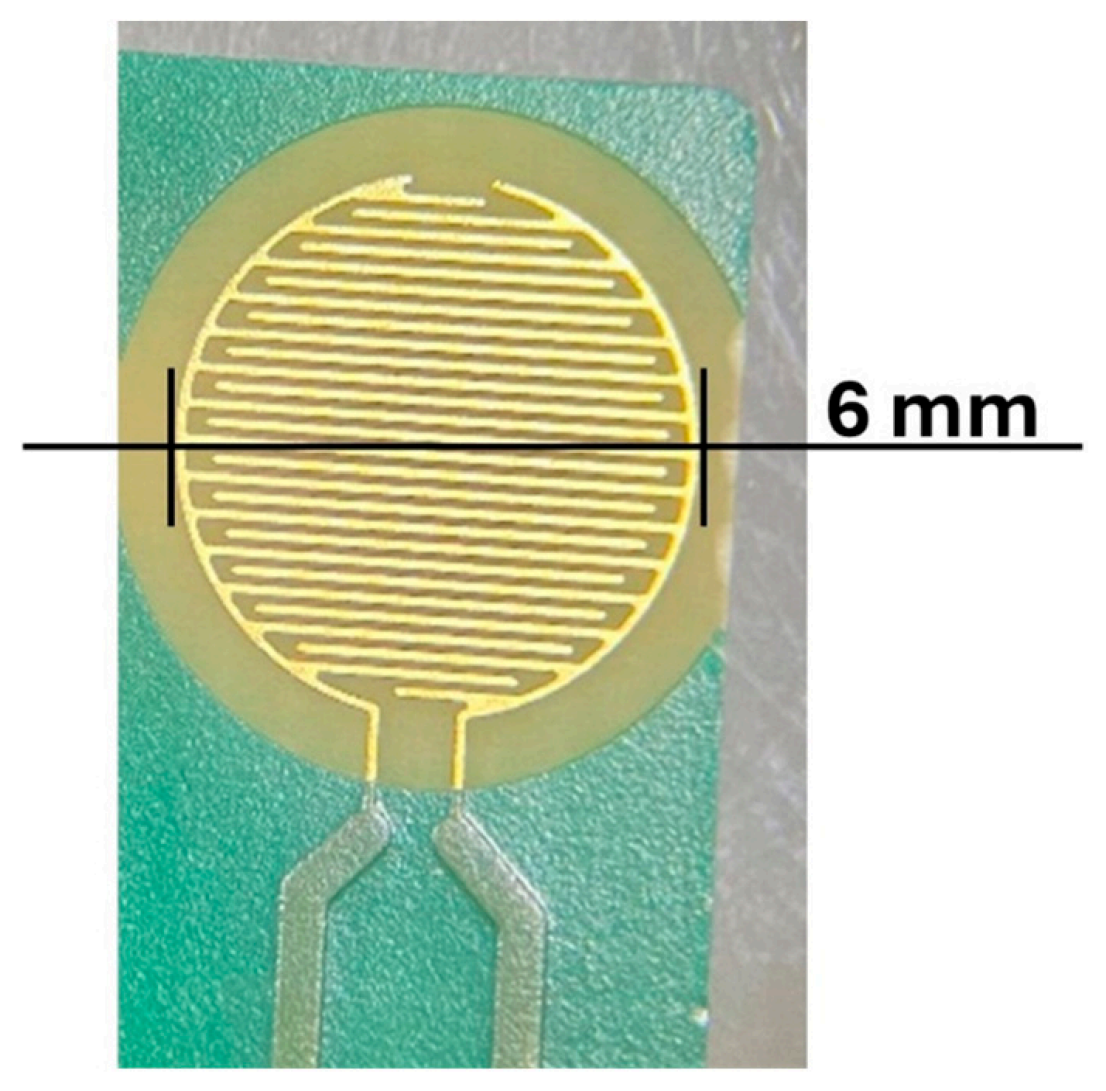
Photo of the microconductometric sensor.

The microconductimetric chip was composed of a pair of interdigitated electrodes deposited through the printed circuit technology on a PCB support. The electrodes were made of a copper layer (42 µm thick) covered with a nickel layer (6 µm) and then with a thin gold layer (120 nm). The electrodes presented a width of 100 µm and an interelectrode distance of 100 µm. The active surface area of the microconductometric sensors is equal to the interelectrode area: 14 mm^2^ (Figure 2).

**Figure 3:**
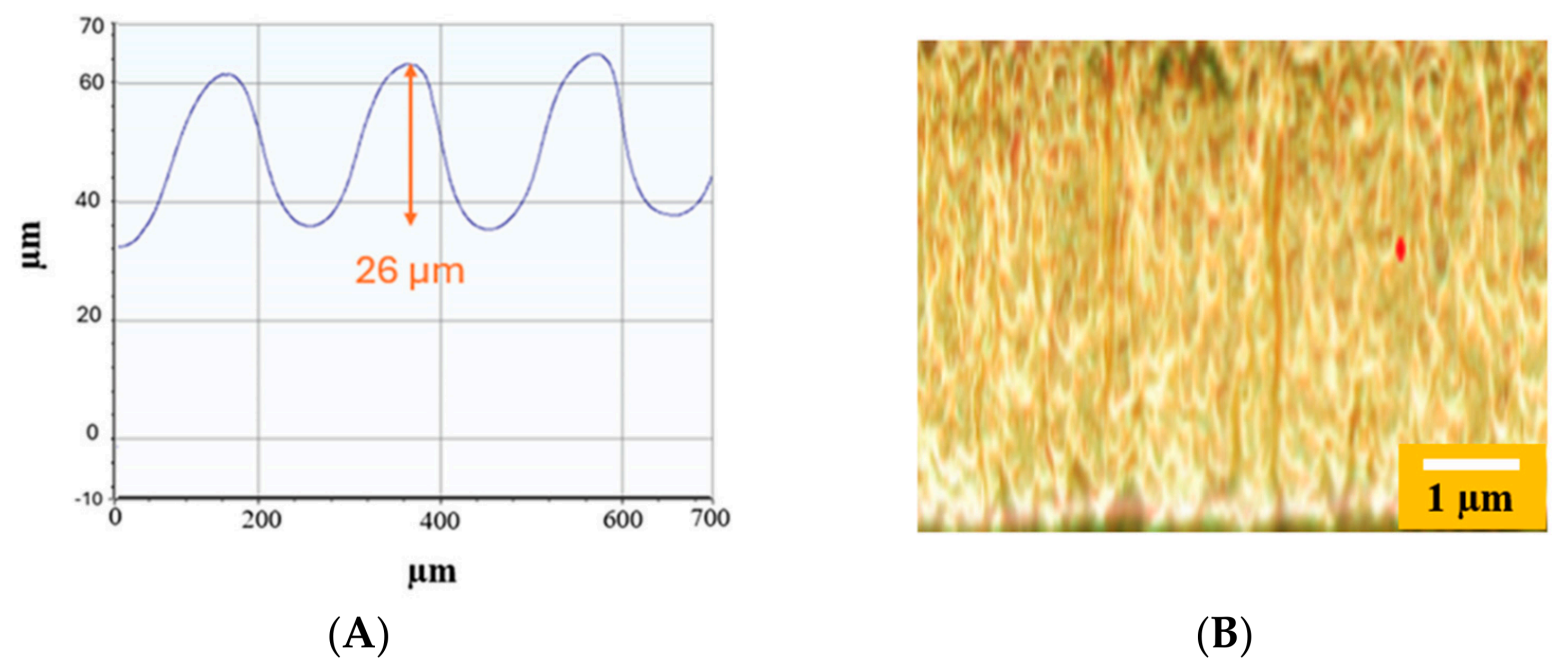
(**A**) Measurements of the PVC membrane thickness on the top of different electrodes; (**B**) SEM observation of the PVC membrane.

The effect of the ion-pair complex content in the membrane was already optimized in previously published papers [24,25]. The same content was kept. The sensing membrane was prepared by dissolving 43 mg of poly(vinyl chloride) (PVC) in 1.5 mL of tetrahydrofuran (THF). Subsequently, 90 mg of bis(2-ethylhexyl) sebacate (DOS), used as a plasticizer, and 10 mg of the synthesized [*o*-COSAN]⁻/glyphosate ion-pair complex were incorporated into the solution under continuous stirring until a homogeneous membrane cocktail was obtained. Aliquots of 5 μL were deposited twice onto the surface of the microconductometric chip: after each deposition, air-drying for one hour was required. The final membrane thickness was 25.0 ± 0.5 µm (Figure 3A). The surface morphology was obtained by SEM observation (Figure 3B), showing its homogeneity. Before use, the fabricated sensor was conditioned in a 10⁻^2^ M glyphosate solution prepared in 10⁻^3^ M phosphate buffer (pH 3.5).

A 10 mV sinusoidal voltage was applied between the pair of interdigitated electrodes, at an optimized frequency of 10 kHz, using a VigiZMeter conductometer (Covarians, Gif-sur-Yvette, France). The conductance measurement was registered online when different concentrations of glyphosate were injected into phosphate buffer, pH 3.5.

## 3. Results and Discussion

### 3.1. FTIR Analysis of the PVC and the Synthesized [o-COSAN]⁻/Glyphosate Ion-Pair Complex

**Figure 4:**
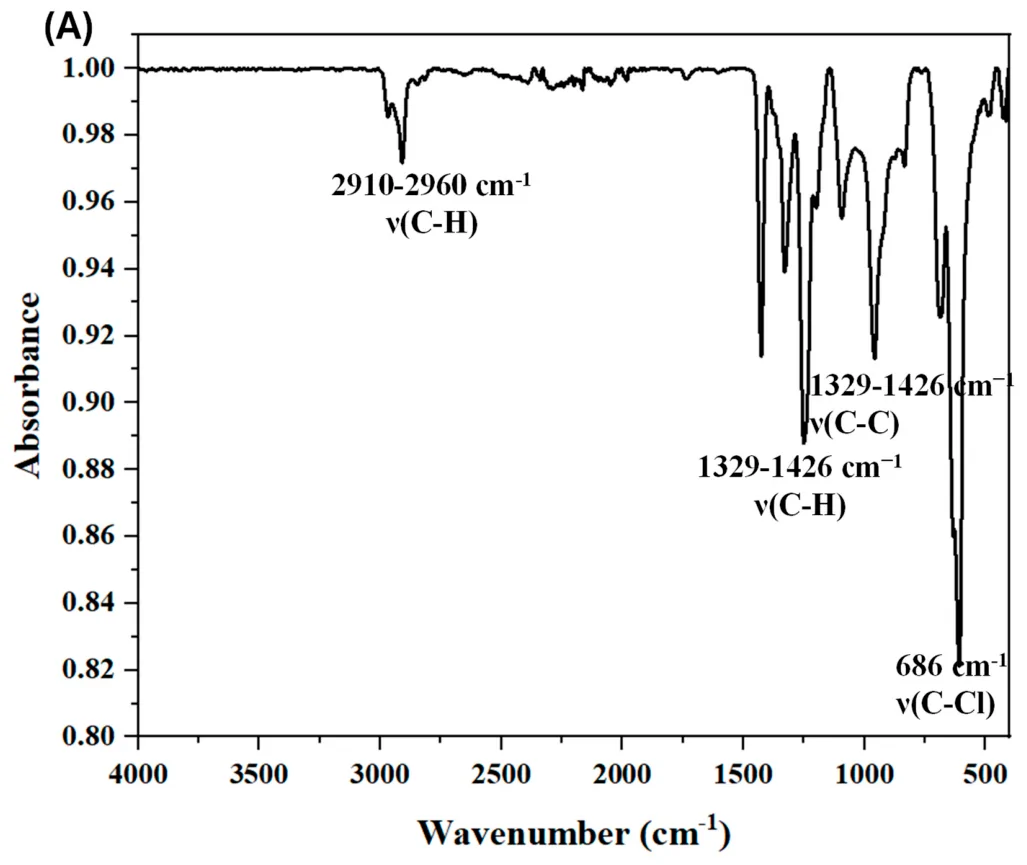

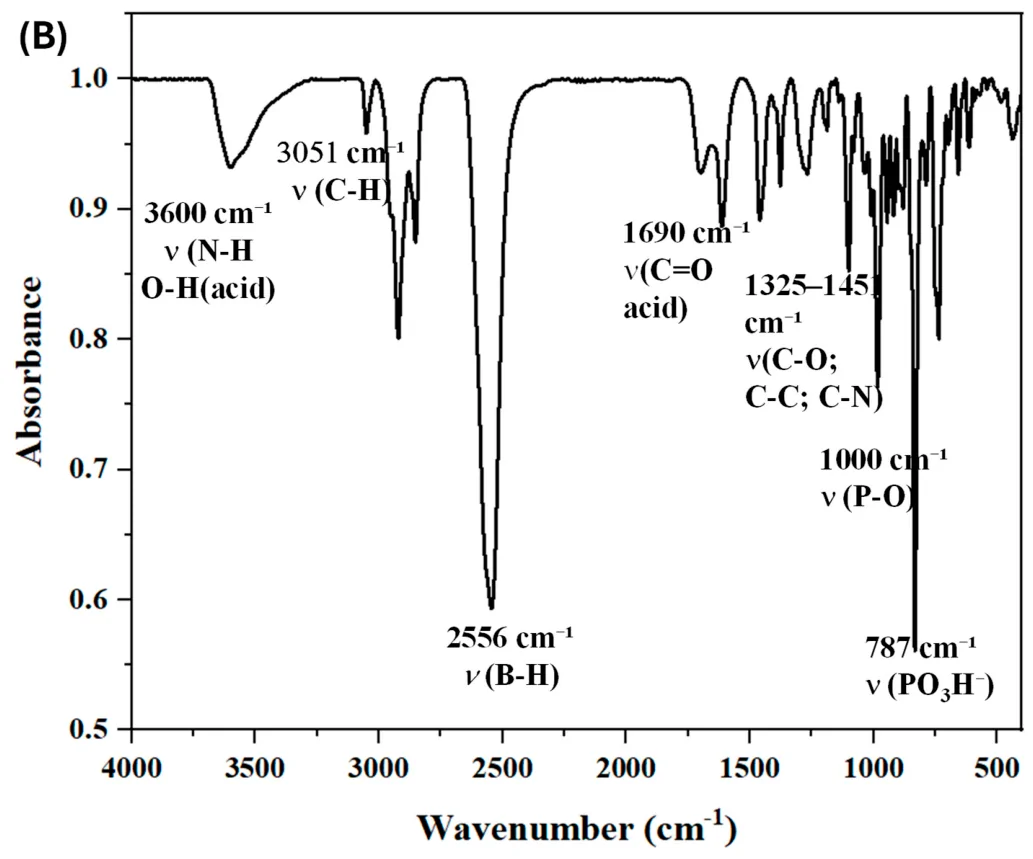
(**A**) FTIR spectrum of PVC. (**B**) FTIR spectrum of [*o*-COSAN]⁻/glyphosate ion-pair complex.

Figure 4A presents the FTIR spectrum of PVC that displays the ν(C-H) at 2910–2960 cm^−1^; ν(C-H) at 1329–1426 cm^−1^; ν(C-C) at 1093–1250 cm^−1^; ν(C-Cl) at 686 cm^−1^.

Figure 4B presents the FTIR spectrum of the prepared [*o*-COSAN]⁻/glyphosate ion-pair complex. One of the most distinctive spectral features is the absorption band located at 2556 cm⁻^1^, corresponding to the ν(B–H) stretching vibration characteristic of the [*o*-COSAN]⁻ cluster. Because this region is typically devoid of absorption from common organic functional groups, this band provides clear evidence for the presence of the boron cluster. Another prominent feature is the broad band centered near 3600 cm⁻^1^, which is assigned to the stretching vibrations of protonated N–H and acidic O–H groups, indicating the incorporation of protonated amino and hydroxyl functionalities into the complex. The band observed at 3051 cm⁻^1^ corresponds to C–H stretching vibrations, whereas the intense peak at 1690 cm⁻^1^ is attributed to the C=O stretching vibration of the carboxylic acid group. Several bands appearing in the 1325–1451 cm⁻^1^ region are associated with C–O, C–C, and C–N stretching modes. The phosphate moiety is identified by characteristic P–OH stretching vibrations at 1188 and 1268 cm⁻^1^, together with P–O stretching bands located at 1000 and 833 cm⁻^1^. Furthermore, the absorption bands at 1268 and 787 cm⁻^1^ can be assigned to the PO_3_H⁻ group, while the signals between 737 and 983 cm⁻^1^ are attributed to C–H deformation vibrations. The presence of these characteristic absorption bands confirms the successful incorporation of glyphosate into the [*o*-COSAN]⁻-based ion-pair complex while preserving the structural features of both components.

### 3.2. Analytical Performance of the Microconductometric Sensor

**Figure 5:**
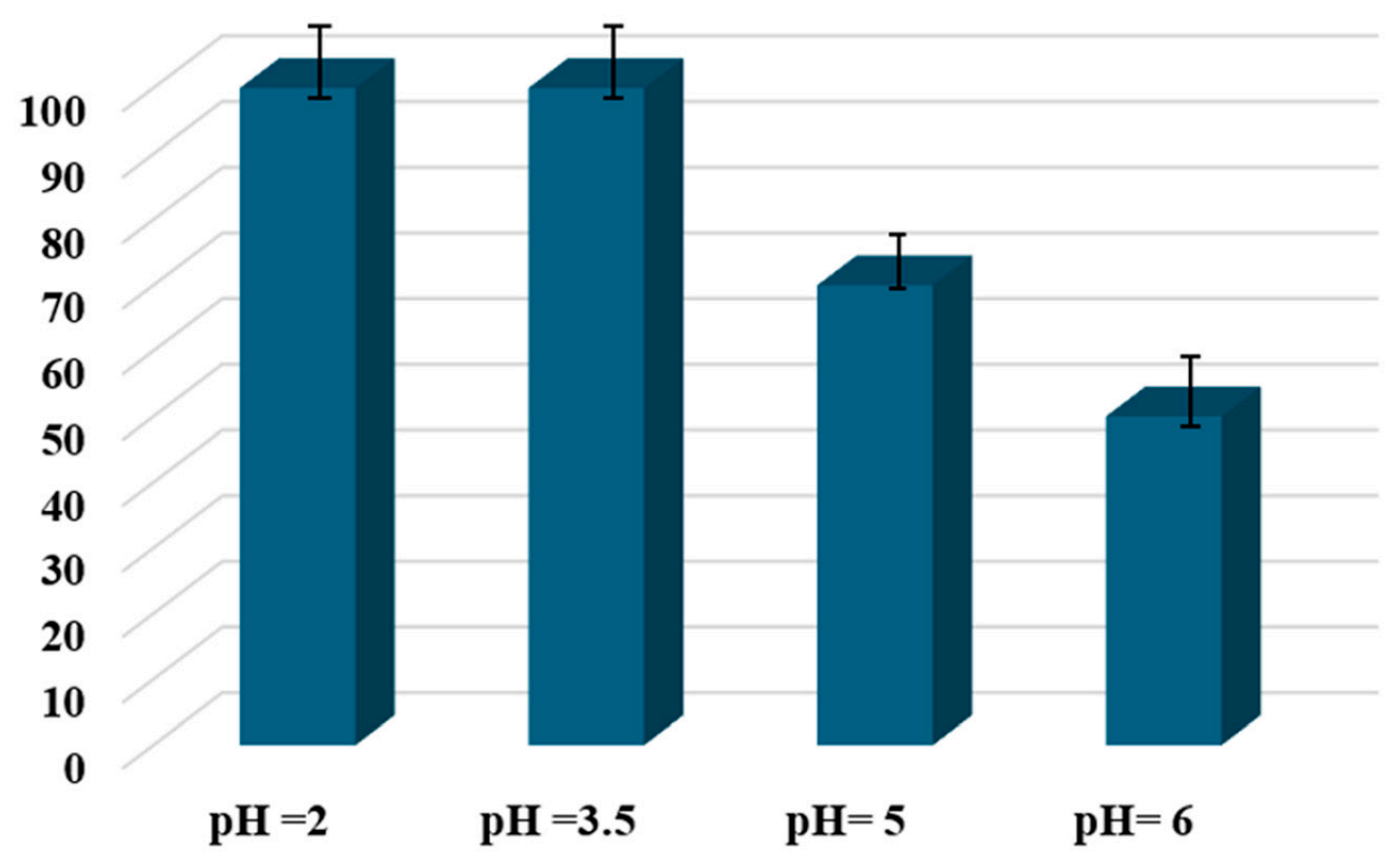
Effect of pH value on the response in conductivity (glyphosate concentration: 100 µM).

The effect of pH on the sensitivity of detection of glyphosate (100 µM) was studied (Figure 5). For pH values equal and lower than 3.5, the signal is high (5.45 µS); it decreases for pH values higher than 3.5, due to the negative charge of glyphosate.

**Figure 6:**
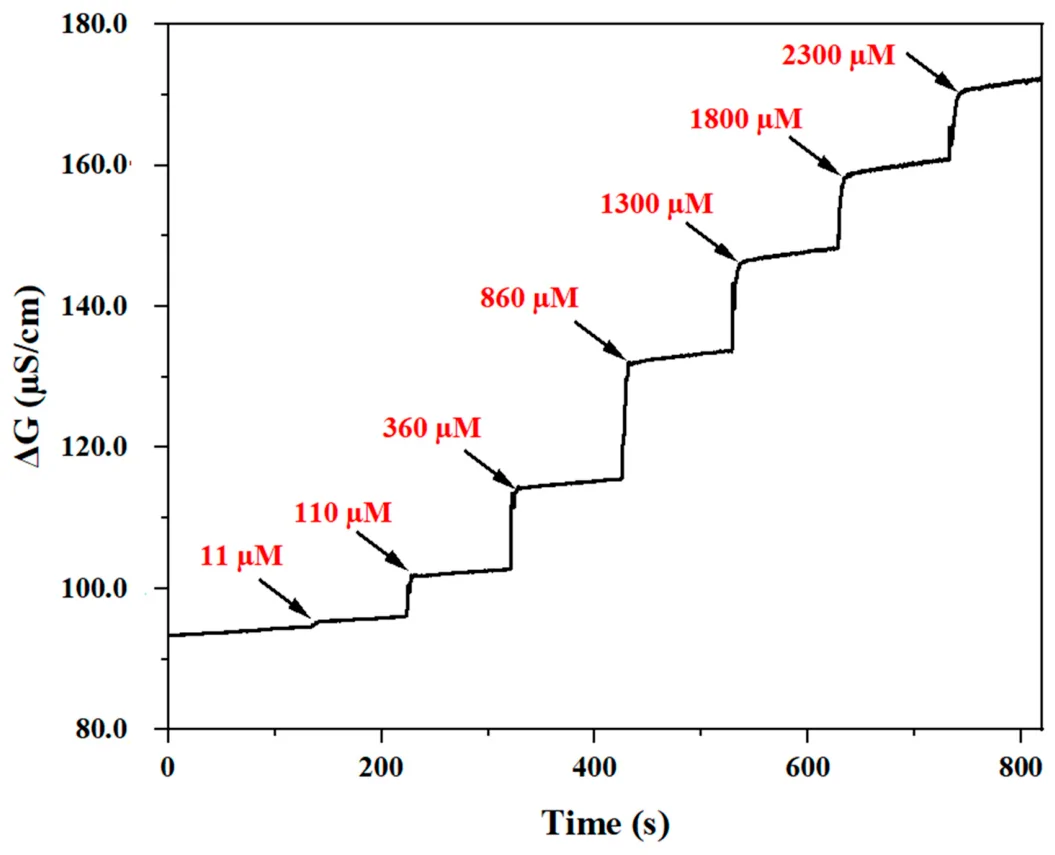
Variation in conductance versus time, when different concentrations of glyphosate are injected into phosphate buffer pH 3.5.

As shown in Figure 6, successive additions of glyphosate into a phosphate buffer solution (pH 3.5) produced a progressive increase in the conductance signal, confirming the effective interaction between glyphosate and the sensing membrane. The sensor exhibited a rapid response, reaching 90% of the steady-state signal within 15 ± 2 s, which demonstrates its suitability for fast analytical measurements. This conductometric response can be attributed to the ion-exchange process occurring at the membrane interface, where the protonated glyphosate molecules interact with the [*o*-COSAN]⁻/glyphosate ion-pair complex, leading to a measurable variation in membrane conductance.

**Figure 7:**
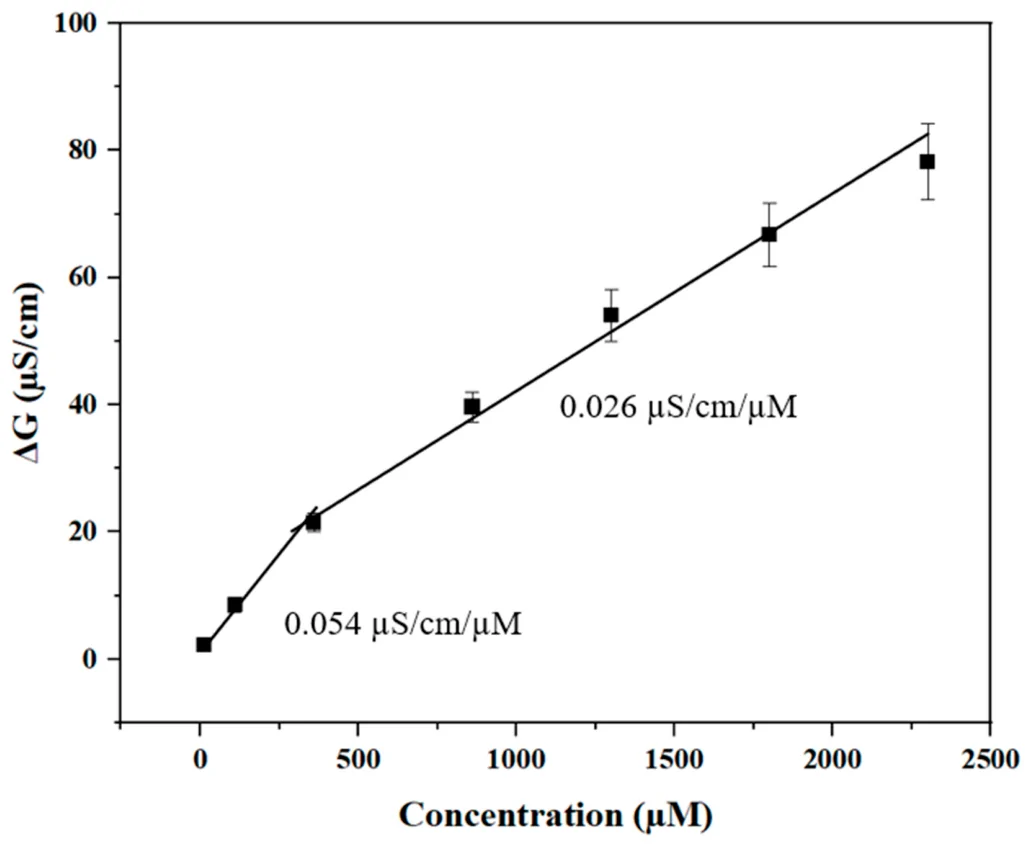
Calibration of the microconductometric sensor for glyphosate.

The corresponding calibration curve is presented in Figure 7. Two distinct linear response regions were observed, reflecting different sensitivities over the investigated concentration range. In the lower concentration domain (11–360 µM), the sensor exhibited a sensitivity of 0.054 µS cm⁻^1^ µM⁻^1^, while a lower sensitivity of 0.026 µS cm⁻^1^ µM⁻^1^ was obtained for concentrations between 360 µM and 2.3 mM. When the concentration exceeds 360 µM, the slope of the calibration curve decreases. This phenomenon is due to the decrease in Donnan exclusion potential, which decreases the permselectivity of the membrane when the concentration of glyphosate increases [26]. The same shape of calibration curve was obtained for the detection of tetracycline with a PVC/[*o*-COSAN]⁻/tetracycline membrane [24], the charge-transfer resistance increasing when the concentration of tetracycline increases, and the slope of the calibration curves decreases when the concentration is higher than 0.1 ng/L.

The detection and quantification capabilities of the suggested sensor were evaluated to evaluate its analytical performance. The limit of quantification (LOQ) was assessed to be 11 μM, while the limit of detection (LOD) was determined to be 4 μM using the 3σ/S criterion (where S is the calibration curve’s slope and σ is the blank signal’s standard deviation). The device exhibits excellent analytical performance, with reproducibility of 3% for 5 measurements of the same concentration with the same sensor and repeatability of 8% (RSD) for the same concentration with 5 different sensors. These results demonstrate the high sensitivity of the developed microconductometric sensor and confirm its suitability for the reliable determination of glyphosate over a broad concentration range, making it a promising platform for environmental monitoring and routine water quality analysis.

The analytical performance of the developed conductometric sensor was benchmarked against previously reported glyphosate sensing platforms, as summarized in Table 1. The reported methods employ a variety of recognition materials, including molecularly imprinted polymers (MIPs), reduced graphene oxide (rGO), ZnTMIPP-based composites, and laser-induced graphene (LIG), combined with electrochemical detection techniques such as EIS, DPV, CV, and LSV.

The comparison reveals that nanostructured electrochemical sensors generally achieve detection limits in the pico- or nanomolar range because of their enhanced electrocatalytic activity and large effective surface area. In contrast, the conductometric sensor proposed in this work exhibits a detection limit of 4 µM over a linear working range of 10 µM–2.5 mM. Despite its reduced sensitivity in comparison to certain nanomaterial-based sensors, the system remains entirely adequate for the determination of glyphosate in contaminated water samples, as evidenced by the recovery studies.

**Figure 8:**
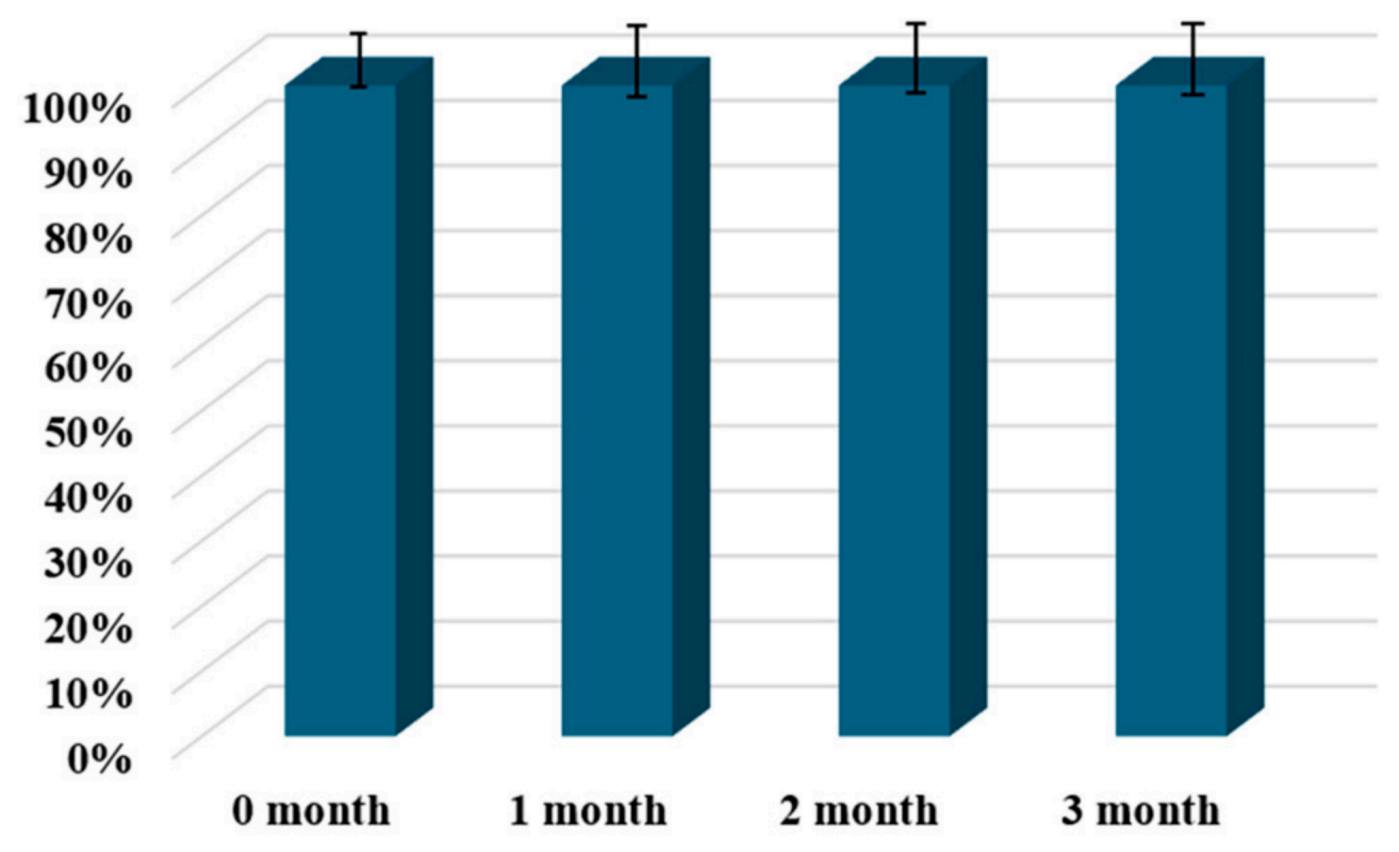
Variation in the response of the microconductometric sensor for three months (glyphosate concentration: 100 µM).

An important advantage of the present sensing platform lies in its operational simplicity. Unlike many previously reported sensors, its fabrication does not require nanomaterial synthesis, sophisticated electrode functionalization, or complex instrumentation. The sensor also provides a rapid analytical response (15 ± 2 s) and good storage stability over at least three months (Figure 8). The variation in the signal was: 5.45 µS for month 0 and month 1 and 5.29 for month 2 and 5.18 for month 3. These characteristics significantly enhance its practicality for routine analyses.

Overall, the comparison highlights that the proposed [*o*-COSAN]⁻/glyphosate ion-pair conductometric sensor offers a balanced compromise between analytical performance and ease of implementation. Its low-cost fabrication, fast response, satisfactory detection capability, and excellent selectivity make it a promising candidate for environmental water quality monitoring, particularly in applications where rapid and portable analysis is preferred over ultratrace detection.

The selectivity of the developed microconductometric sensor was evaluated by investigating its response toward potential interfering compounds commonly encountered in environmental samples. Two representative interferents were selected: aminomethylphosphonic acid (AMPA), the major degradation product of glyphosate, and carbofuran, a widely used pesticide with a chemical structure different from glyphosate. Each compound was individually injected at a concentration of 50 µM under the same experimental conditions used for glyphosate detection.

**Figure 9:**
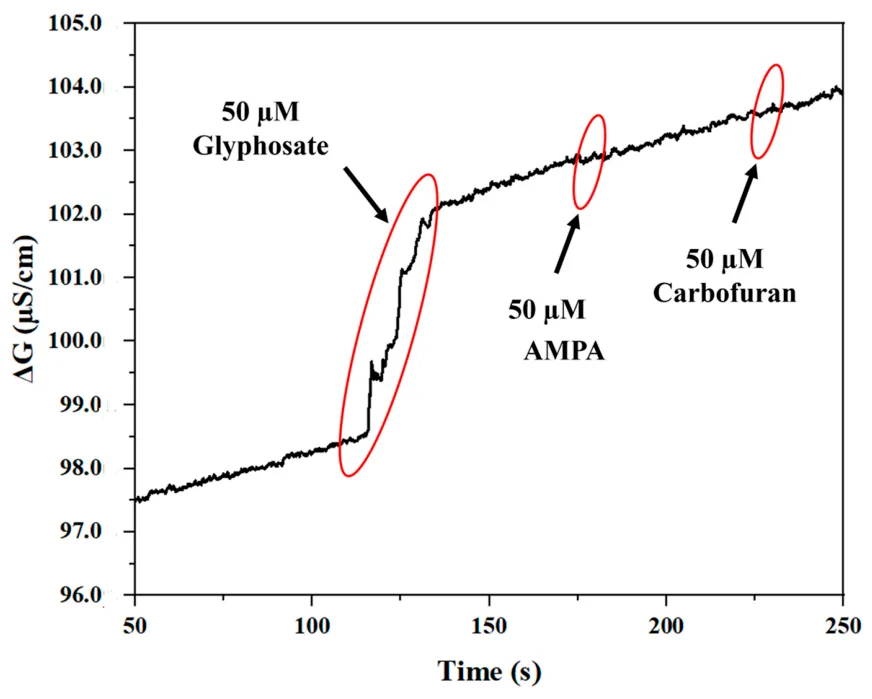
Response of the microconductometric sensor to 50 µM injections of glyphosate, aminomethylphosphonic acid (AMPA), and carbofuran.

As illustrated in Figure 9, the injections of both AMPA and carbofuran produced no significant variation in conductance, whereas glyphosate generated a clear and reproducible sensor response. These results demonstrate that the sensing membrane exhibits a high affinity toward glyphosate while effectively discriminating against structurally related and unrelated compounds. The absence of measurable interference confirms the excellent selectivity of the [*o*-COSAN]¯/glyphosate ion-pair complex, indicating that the recognition mechanism is highly specific to glyphosate.

The high selectivity of the proposed sensor is particularly advantageous for environmental monitoring, where glyphosate is often present together with its degradation products and other agrochemicals. This feature minimizes the risk of false-positive responses and contributes to the reliability of glyphosate quantification in complex water matrices.

### 3.3. Real Sample Analysis

The practical applicability of the developed microconductometric sensor was assessed through the determination of glyphosate in two representative environmental matrices, namely tap water and river water. Before analysis, these samples were filtered through a 0.45 µm filter, and then the pH of both samples was adjusted to 3.5 to ensure optimal sensing conditions for glyphosate. The acidification process could change the speciation of the compounds in these samples, but for glyphosate, only its charge value could change. The water samples were then spiked with glyphosate at two concentration levels (100 µM and 500 µM), representing both low and relatively high contamination scenarios.

The obtained results, summarized in Table 2, demonstrated excellent analytical performance, with an average recovery of 102%. These recovery values indicate that the sensor provides accurate and reliable quantification of glyphosate in both water matrices, with negligible interference from the sample composition. The close agreement between the added and measured concentrations confirms the robustness of the sensing platform and highlights its capability to operate effectively in real environmental samples without significant matrix effects.

These findings demonstrate the suitability of the proposed microconductometric sensor for practical environmental monitoring. Its satisfactory recovery, combined with its rapid response, simple operation, and low-cost fabrication, makes it a promising analytical tool for routine glyphosate determination in surface and drinking water samples.

## 4. Conclusions

A microconductometric sensing platform was designed using a PVC membrane incorporating an [*o*-COSAN]⁻/glyphosate ion-pair complex for the selective determination of glyphosate. The fabricated sensor exhibited a rapid response time (15 ± 2 s), together with a detection limit of 4 μM and a wide linear response range, demonstrating analytical performance comparable to that of more sophisticated electrochemical sensing platforms. Furthermore, the sensor showed excellent selectivity toward glyphosate over its main metabolite (AMPA) and another pesticide, carbofuran, confirming the effectiveness of the ion-pair complex as a recognition element. The membrane also exhibited good stability, maintaining its performance for at least three months at room temperature.

Beyond its analytical performance, the proposed sensing platform offers important practical advantages, including simple fabrication, low cost, and suitability for rapid analysis. These characteristics make it a promising alternative for routine glyphosate monitoring in environmental samples. Moreover, the [*o*-COSAN]¯/glyphosate ion-pair membrane could be readily integrated into other electrochemical transducers, such as ion-selective electrodes (ISEs) and ion-sensitive field-effect transistors (ISFETs), broadening its potential applications. Future work will focus on improving the sensitivity of the sensor and validating its performance in real environmental water samples to support practical environmental monitoring.

## Figures and Tables

**Table 1:** Comparison of the developed sensor for the detection of glyphosate to those of reported nanomaterial-based sensors in the literature.

Recognition Part	Techniques	Range of Detection (M)	Limit of Detection (M)	Ref.
MIP chitosan	EIS	1.8 pM–0.3 mM	30 fM	[15]
MIP polypyrrole/nanotubes	DPV	14.6 nM–2.07 mM	11.2 nM	[16]
rGO	DPV	1–100 nM	0.144 nM	[17]
ZnTMIPP/ITO	CV	10 nM–10 µM	4.14 nM	[18]
laser-induced graphene (LIG)	LSV	5–1000 µM	9 µM	[19]
[*o*-COSAN]⁻/glyphosate ion-pair complex	conductometry	10 µM–2.5 mM	4 µM	This work

**Table 2:** Application of the developed sensor for glyphosate detection in water samples.

	Added (µM)	Found (µM)	Recovery (%)
Tap water	100	99.9	99 ± 3
	500	510	102 ± 3
River water	100	100.2	102 ± 3
	500	515	103 ± 3

## Data Availability

The data that support the findings of this study are available from the corresponding author upon reasonable request.
